# Polymer-Induced Drag Reduction in Dilute Newtonian and Semi-Dilute Non-Newtonian Fluids: An Assessment of the Double-Gap Concentric Cylinder Method

**DOI:** 10.1021/acs.iecr.2c00899

**Published:** 2022-07-20

**Authors:** Stefanos Michaelides, Kotaybah W. Hashlamoun, Thibaut Charpentier, Gregory de Boer, Paul Hunt, Helen Sarginson, Claire Ward, Nashaat N. Nassar, Mark C. T. Wilson, David Harbottle

**Affiliations:** † School of Chemical and Process Engineering, University of Leeds, Leeds LS29JT, U.K.; ‡ Department of Chemical and Petroleum Engineering, University of Calgary, Calgary, Alberta T2N1N4, Canada; § School of Mechanical Engineering, University of Leeds, Leeds LS29JT, U.K.; ∥ CRODA Europe Ltd., Goole DN149AA, U.K.

## Abstract

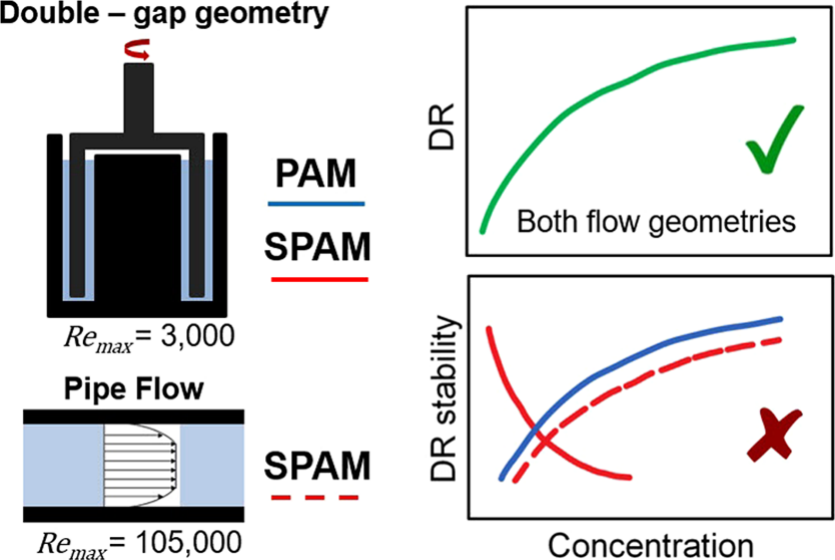

Polymer-induced drag reduction (DR) in fluids was studied using a rotational rheometer with double-gap concentric cylinder geometry. Although both polymers (polyacrylamide (PAM) and 2-acrylamido-2-methylpropane sulfonic acid (SPAM)) had molecular weights of several MDa, the contrasting polymer charge, nonionic and anionic, led to different polymer overlap concentrations (c*), PAM ≫ SPAM, and fluid rheology, with PAM fluids mostly Newtonian and SPAM fluids non-Newtonian (shear-thinning). Based on these differences, it was important to account for the infinite shear viscosity and normalize the polymer concentration by the intrinsic concentration (*c*_int_) so that the DR performance of the two polymer fluids could be accurately compared. Both polymers induced DR, and the maximum DR by SPAM (DR% = 28) was slightly higher than that by PAM (DR% = 22) when *Re*_p_ ∼ 1700. For PAM, the loss of DR with time diminished at higher polymer concentrations (≥100 ppm, at *Re*_p_ = 3149) but was found to be sensitive to high *Re*_p_, with polymer chain scission the likely cause of the reduced performance. For the semi-dilute SPAM fluids, the shear stability contrasted that of PAM, showing negligible dependence on the polymer concentration and *Re*_p_. The apparent rapid loss of DR was predominantly attributed to a time-dependent effect and not polymer degradation. In pipe flow, the maximum DR for SPAM was higher than that measured by rheometry and was attributed to differences in the flow conditions. However, changes in the normalized DR/c with polymer concentration were found to be consistent between the two flow geometries. Furthermore, the high fluid stresses in pipe flow (at high *Re*_p_) led to drag reduction losses consistent with PAM, as the time-dependent effect was not seen.

## Introduction

With global energy demand continuing to increase, significant effort must be made to improve the efficiency of energy-intensive systems and processes. In the pumping of fluids, frictional drag costs energy, which can be reduced by adding very low concentrations of soluble high-molecular-weight polymers to the fluid.^1−8^ Polymer drag reduction (DR) has found application in fluid transport in pipelines,^9^ hydrofracking,^10,11^ flows in heat exchangers,^12^ fire-fighting equipment^13^ and medicine.^14^ The Trans-Alaskan pipeline is one of the largest demonstrations of polymer-induced drag reduction, with the pipeline pressure drop reduced by 80% when adding low concentrations (∼100 ppm) of a very high-molecular-weight polymer.^15^

Since the early work of Toms,^16^ significant progress has been made in understanding the governing principles of polymer drag reduction. It is now widely accepted that drag reduction can be induced using polymers of sufficiently high molecular weight, added at concentrations above a critical level, and when a minimum level of turbulence intensity is achieved in the flow.^1,17,18^ The two theories of polymer drag reduction are: (i) viscous theory, which describes the effect of an increased fluid viscosity near the pipe wall, increasing the buffer layer thickness and suppressing turbulent fluctuations; and (ii) elastic theory, which assumes a negligible increase in the effective viscosity. The latter proposes that the buffer layer thickness increases when the elastic energy stored by the polymer chains is similar to the kinetic energy in the buffer layer at a given length scale greater than the Kolmogorov scale, arguing that the so-called Kolmogorov energy cascade is interrupted. As a result, eddy length scales below the Kolmogorov scale start to behave elastically.^19,20^

Pipe and duct flows have been used to study polymer drag reduction, with changes in the pressure drop or velocity profile providing a direct measure of performance.^19,21−23^ However, such methods are often costly, time-consuming and require large volumes of fluid; thus, the approach is not best-suited to rapidly screen polymers. Rotational methods measure drag reduction by comparing the torque difference between the polymeric solution and the solvent at varying rotational velocities. One of the first reported studies by Choi and Jhon,^24^ used a rotating steel disk in a cylindrical container to determine the drag reduction of poly(ethylene oxide) (PEO) and polyisobutylene (PIB). A more commonly used geometry is a concentric cylinder, where shear-driven Taylor–Couette flow occurs at high rotational speeds.^25−27^ With a high surface area, the geometry enables detection of drag reduction at a lower Reynolds number (*Re*), and with a precision that allows small differences in performance to be meaningfully interpreted. For high rotational velocities and small annular gap size, the boundary layer at the wall can be sufficiently described by the Prandtl–von Karman equation, similar to other canonical flows such as a wall-bounded pressure-driven flow.^28,29^ In such shear-driven rotational flows, the polymer acts to modulate Taylor instabilities which are a function of the rotational speed and fluid viscoelasticity.^30^

Rajappan and McKinley^31^ modified the size of the rotor–stator of a TA Instruments AR-G2 rheometer to increase the gap size, thus accessing a higher *Re* range to study the drag reduction performance of an extracted polysaccharide in featureless turbulence. This is one of only a few studies that compared performance in the regime of featureless turbulence, but when combined with the rheometer, provides a level of sensitivity often unattainable using bespoke instruments. However, most studies only consider drag reduction in flow regimes in the absence of featureless turbulence.

The drag reduction performance of high-molecular-weight polymers such as water-soluble PEO, polyacrylamide (PAM), and xanthan gum have been frequently studied using concentric cylinder geometries. Such polymers are of interest because of their contrasting intrinsic properties, for example, xanthan gum is a rigid polymer that exhibits type B drag reduction, resulting in the early onset of drag reduction (so-called retro-onset, at low turbulence), and the magnitude of drag reduction is almost independent of *Re*. This contrasts type A polymers where the drag reduction is a function of *Re*, as the polymer coils are stretched at higher turbulence intensity.^32^ Generally, the shear stability increases in the order PEO < PAM, with the better performance of PAM not fully understood but likely attributed to the greater polymer rigidity and its resistance to polymer chain scission.^33,34^ Often, the loss of drag reduction can be attributed to polymer chain scission, although de-aggregation of polymers has also been noted to impart time-dependent effects.^35^

In the current study, the drag reduction performance of dilute polyacrylamide (PAM) and semi-dilute 2-acrylamido-2-methylpropane sulfonic acid (SPAM) polymers have been assessed using rotational rheometry. The rheometer method has frequently been used to determine the drag-reducing properties of many polymers, yet the flow conditions attained are often limiting when compared to pipe flow. The study brings attention to the methods that should be used to correctly interpret the drag reduction performance of high viscosity and semi-dilute polymer samples and directly compares performance with that observed in pipe flow. While trends of DR% with polymer concentration are consistent, significant differences in DR stability were observed and could be attributed to a time-dependency effect of the SPAM polymer that is more apparent when the fluid stresses are lower.

## Materials and Experimental Methods

The polyacrylamide with ∼30% sulfonation (2-acrylamido-2-methylpropane sulfonic acid, SPAM) and with a manufacturer-quoted molecular weight between 5 and 8 × 10^6^ Da was provided by SNF Floerger (France). The nonionic polyacrylamide (PAM) with a quoted molecular weight of 5 × 10^6^ Da and potassium chloride (99.0% pure, KCl) were supplied by Sigma-Aldrich, U.K. All chemicals were used without further purification. The water used in the study was deionized water (Milli-Q) with a resistivity of 18.2 MΩ cm at 25 °C.

### Polymer Preparation

A polymer stock solution of 10,000 ppm was prepared by adding the required amount of dry polymer powder into Milli-Q water (unadjusted pH) at room temperature. The polymer was then dissolved by gently mixing the solution on a lab roller for 48 h, with complete dissolution visually assessed. The polymer solution was stored at room temperature in a sealed glass vial, and the solution was used within 30 days of its preparation. The sample was not observed to degrade during this time. Prior to use, the concentrated polymer solution was gently mixed overnight using a magnetic stirrer. Samples were then removed from the glass vial and diluted with Milli-Q water to the desired concentration for rheology assessment. For certain tests, a monovalent electrolyte KCl was added following the dilution step, and the polymer solutions were left to gently agitate for 2 h to ensure the samples were homogeneous prior to measurement.

### Rheology

**Figure 1:**
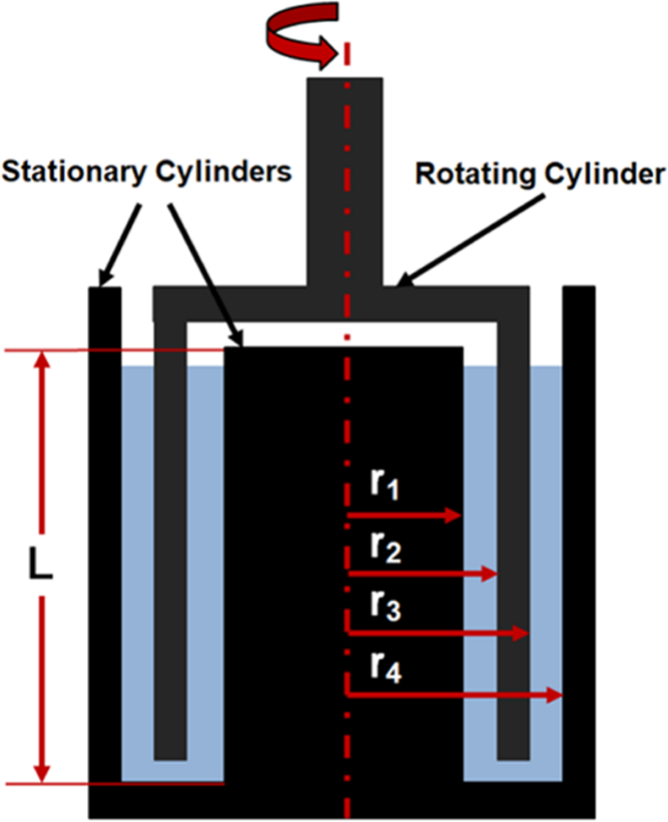
Schematic representation of the double-gap coaxial cylinder used to measure the rheology and drag reduction performance of the polymeric fluids.

A DHR-II rheometer (TA Instruments, U.K.) was used to measure the sample shear viscosity over a shear rate range of 0.01–500 s^–1^. The geometry used was a double-gap concentric cylinder, as shown in Figure 1, which has dimensions of *L* = 55 mm, *r*_1_ = 15.1 mm, *r*_2_ = 16 mm, *r*_3_ = 17.5 mm, and *r*_4_ = 18.5 mm. The instrument was first calibrated following the standard protocol to determine the inertia of the rotor and geometry and to ensure the geometry was lowered to a gap distance of 2000 μm. The sample volume of 11 mL was gently transferred to the cup using a wide bore pipette before lowering the geometry to the standard gap. All measurements were conducted at 25 (±0.1) °C, with the temperature maintained using a Peltier jacket. At each desired shear rate, the fluid viscosity was measured after 5 s equilibration time and averaged over 30 s measurement time.

### Fluid Drag Reduction

The DHR-II rheometer with the double-gap geometry (Figure 1) was used to measure the drag reduction performance of the polymeric fluids relative to Milli-Q water. The same instrument calibration procedure as described previously was followed, and all measurements were conducted at 25 (±0.1) °C. The drag reduction performance was studied to determine the magnitude of drag reduction, measured from a flow sweep test, and the stability of drag reduction, measured from a peak hold test. No pre-shear protocol was used. Once the sample was added to the rheometer cup, the geometry was lowered to the gap setting, the solvent trap was added, and the sample was left undistributed for 2 min prior to measurement to ensure thermal equilibrium was attained. For the flow sweep test, the rotational speed (ω) of the geometry was increased from 3 to 200 rad/s using a logarithmic ramp collecting 100 data points over a test duration of 420 s. The relatively fast flow sweep test was chosen to minimize possible sample degradation during the shear ramp but was slightly compromised by the need to have an equilibration time of 2 s to attain a steady state before a measurement time of 2 s at every predetermined rotational speed. For the peak hold test, the sample preparation and sample loading followed the same standard protocol. However, no shear ramp was used, and the polymer solution was almost instantaneously sheared at a range of rotational speeds between 100 and 200 rad/s with the instrument torque measured every 2 s for 1000 s to follow the transient behavior. All experiments were completed in triplicate and were found to be reproducible within ±2%.

### Data Analysis

For rheometry, the torque (*M*, μN m) on the rotating double-gap coaxial cylinder is related to the shear stress (τ, Pa) through the stress constant (*k*_τ_) of the geometry (, where *c*_L_ is the geometry aspect ratio) such that
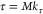
1

Assuming a smooth surface, the shear stress can be transformed into a Fanning friction factor by^27^
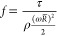
2where ρ is the fluid density (997 kg/m^3^), ω is the rotational velocity (rad/s), and , with *r*_2_ and *r*_3_ as previously defined. The Reynolds number (*Re*) is used to represent the flow as a dimensionless value and taken to be the ratio of inertial to viscous forces^27,28^
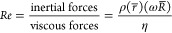
3where *r̅* = ((*r*_2_ – *r*_1_) + (*r*_4_ – *r*_3_))/2 and η (Pa s) is taken as the infinite shear viscosity of the fluid. In the following text, *Re*_s_ and *Re*_p_ describe the shear Reynolds numbers of the solvent and polymer fluid, respectively. The fluid drag reduction is calculated by
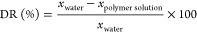
4where *x*_water_ and *x*_polymer solution_ are either the torque at an equivalent rotational velocity or the Fanning friction factor at an equivalent *Re* for the pure solvent (deionized water) and polymer fluid, respectively.

### Pipe Flow

A 51 L industrial size friction flow loop (Charlton & Hill Welding LTD, Alberta, Canada) of 18 mm I.D. (stainless steel pipe) was used to measure the polymer-induced drag reduction at a constant flow rate of 80 L/min (*Re*_s_ ∼ 105,000) at 21± 2 °C, with the slight variation in temperature due to no temperature control on the flow loop. All polymer samples were prepared using the standard protocol as previously described. Prior to each test, the pipe loop was flushed with excess water (rinse cycles of 10 min after each run) to remove any residual chemicals. In the current study, tests were run in order of increasing additive concentration so as to minimize error from any residual drag-reducing additive. To begin, the test fluid without polymer was circulated around the flow loop for 30 s until the pressure drop for water reached a steady baseline. Then, concentrated polymer solutions were injected into the feed tank, with the flow rate kept constant to obtain the desired concentrations for each experiment. The fluid was pumped using a progressive cavity pump (TOSHIBA 0106SDSR41A-P), and the flow rate was measured using a Coriolis flow meter. The pressure drop was measured by differential pressure transducers (Stellartech.) separated at a distance (*L*) of ∼7.3 m. The drag reduction was calculated using eq 4, where *x* is Δ*P*. Flow loop tests were run for 40 min at a constant flow rate to study the stability of drag reduction. The data was then processed using the data acquisition software (Siemens SIMATIC WinCC Comfort V14 SP1). All experiments were completed in duplicate and were found to be reproducible within ∼4%.

## Results and Discussion

In rotational shear-driven flows, flow instabilities occur as a function of increasing rotational speed. For the double-gap geometry, the relative difference in radial velocity between *r*_1_ and *r*_2_, and *r*_3_ and *r*_4_ results in nonuniform instabilities, with the outer gap experiencing instabilities at a lower critical *Re* compared to the inner gap. The flow instability is referred to by the Taylor number, which is calculated from the ratio of centrifugal to viscous forces^27^
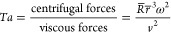
5where *v* is the kinematic viscosity (m^2^/s).

**Figure 2:**
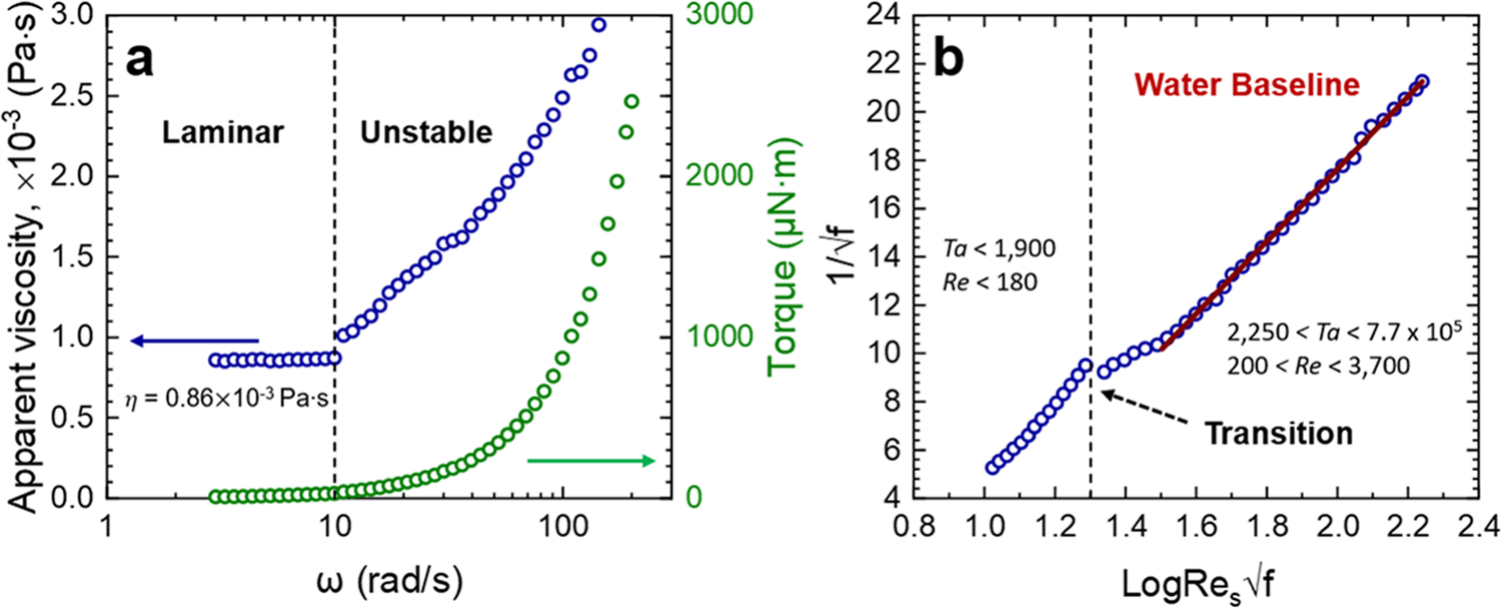
(a) Real and apparent viscosity of Milli-Q water as a function of rotational speed (ω). The laminar flow regime is up to ω ∼ 10 rad/s. (b) Empirical data in panel (a) plotted using Prandtl–von Karman coordinates on a semi-log scale. The solid line takes the form  and the fitting parameters *A* and *B* are 15.014 and −12.38, respectively.

Figure 2a shows typical apparent shear viscosity  data of increasing rotational speed of the geometry and the transition from the laminar (stable) flow regime. The test fluid is Milli-Q water and at low shear rates (ω < 10 rad/s), the fluid is Newtonian, with a viscosity of 0.87 × 10^–3^ (±0.01) Pa s. Beyond ω = 10 rad/s, the apparent fluid viscosity increases, which signifies the onset of secondary flow and Taylor instabilities. The Taylor number at the transition is *Ta* ∼ 1900 and in reasonable agreement with the value reported by Taylor^36^ (for a classical bob and cup geometry with one annular gap), although the number is slightly higher due to contributions from the inner and outer gaps. The measured torque is also shown in Figure 2a and follows a second-order polynomial regression response. The Milli-Q water data is used as a baseline comparison to the polymer fluids.

Figure 2b replots the raw data in Prandtl–von Karman coordinates (semi-log plot), where 1√*f* and Log *Re*_s_√*f* use the Fanning friction factor, as described in eq 2. For rotational speeds up to 200 rad/s, the maximum attainable *Re*_s_ and *Ta* values were 3700 and 7.7 × 10^5^, respectively. The transition to unstable flow is easily identified (highlighted by the dashed line), and with increasing Log *Re*_s_√*f*, the flow regime transitions from wavy to modulated to turbulent Taylor vortices.^28^

Similar to pipe flow, the rheometer data (Figure 2b) can be described by a least-squares linear fit of the form given by eq 6 (where *A* and *B* are geometry-dependent variables) when Log *Re*_s_√*f* ≥ 1.54 (*Re*_s_ ∼ 380).
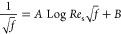
6

Equation 6 is known as the Prandtl–von Karman law,^37^ which describes fully developed turbulence in pipe flow. Its use in the current study is more empirical to provide a method of comparison for the different test fluids. The method provides reasonable data fitting, although the region just after the transition to unstable flow is less well-described by the fit.

**Figure 3:**
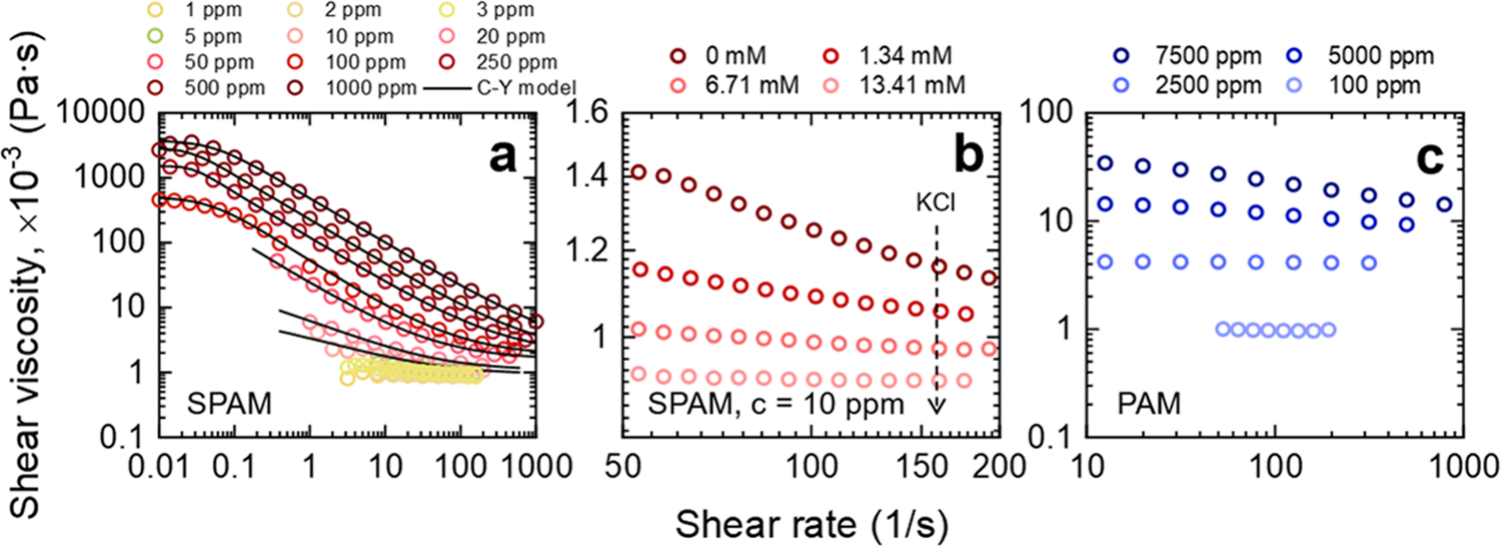
(a) Shear rate-dependent fluid viscosity as a function of polymer type (a, b) SPAM and (c) PAM. The effect of polymer concentration is shown in panel (a) and panel (c), and the effect of electrolyte (KCl) concentration is shown in panel (b). The fits in panel (a) are based on the Carreau–Yasuda model, see eq 7.

Converting the rheometer data to Prandtl–von Karman coordinates is readily achieved for Newtonian fluids; however, the assessment of fluid viscosity is more complicated for non-Newtonian fluids, such as those encountered in the current study; see Figure 3. The viscosity of the SPAM fluid strongly depends on the shear rate, confirming its non-Newtonian behavior, with the response dependent on the polymer concentration (Figure 3a) and electrolyte concentration of the base fluid (Figure 3b). A greater degree of shear-thinning was observed for higher polymer concentrations but lower electrolyte concentrations. The effect of polymer concentration on the rheology of nonionic PAM was less significant, with a Newtonian response up to 2500 ppm, and a weakly shear-thinning response at 7500 ppm (Figure 3c). Such contrasting fluid behaviors result from the different polymer conformations. With its strongly charged backbone, SPAM adopts an extended rod-like conformation in water; hence, the polymer chains overlap at much lower concentrations than polymers that adopt a coiled conformation, such as the nonionic PAM. Using the shear viscosity (Figure 3), the polymer chain overlap concentration (c*) for the two polymers was approximated by calculating the specific viscosity, η_sp_. It should be noted that the more accepted method of c* ≈ 1/[η] (where [η] is the intrinsic viscosity measured using a capillary viscometer) was initially considered; however, the reduced viscosity of SPAM with concentration did not vary linearly, and thus it was not possible to reliably determine [η]. Using the method of specific viscosity,^38,39^ the c* for PAM and SPAM was ∼1500 and 0.01 ppm, respectively. For PAM, the c* value was in excellent agreement with that calculated using [η], [η] = 6.5*dLg*^*–*1^, *c*_[η]_* ∼ 1530 ppm. While there may be some error in the reported c* value of SPAM (the value could not be verified from [η]), the non-Newtonian response of SPAM was seen at a significantly lower concentration than that for PAM; therefore, the reported difference in c* values is reasonable. Further details of this method are provided in Figure S1 of the Supporting Information. The high occupied volume and greater interaction between SPAM chains increase the fluid viscosity, but with increased hydrodynamic forces (higher shear rates), the chains disentangle and align with the flow to induce a shear-thinning fluid response.^40^ The charge screening effect in high electrolyte solutions weakens the rigidity of the polymer backbone (induced by charge repulsion) and allows SPAM to adopt a more coiled conformation. It is reasonable to assume that the collapse of the polyelectrolyte due to salt addition will ultimately lead to a conformational state resembling that of its nonionic counterpart (PAM) in a good solvent.^41,42^ Therefore, the weak shear-thinning response at 10 ppm can be diminished when up to 13.41 mM KCl is added to the base fluid (Figure 3b).

To approximate the fluid viscosity in the regime of drag reduction, the approach was taken to fit the flow curve data using the Carreau–Yasuda model to determine the infinite shear viscosity (η_∞_) and then calculate *Re* using eq 3. The Carreau–Yasuda model is given by
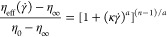
7where η_eff_(γ̇) is the fluid viscosity as a function of shear rate, η_0_ is the zero-shear viscosity, η_∞_ is the infinite shear viscosity, κ is the consistency index, *n* is the power-law index, and *a* describes the transition from Newtonian to power law behavior. Details regarding all parameters of the Carreau–Yasuda model are provided in Table S1 of the Supporting Information. For the nonionic PAM, the fluid was found to be Newtonian or weakly non-Newtonian at the polymer concentrations used for drag reduction testing, thus the η_∞_ was taken to be the fluid viscosity at the highest measured shear rate.

### Drag Reduction

**Figure 4:**
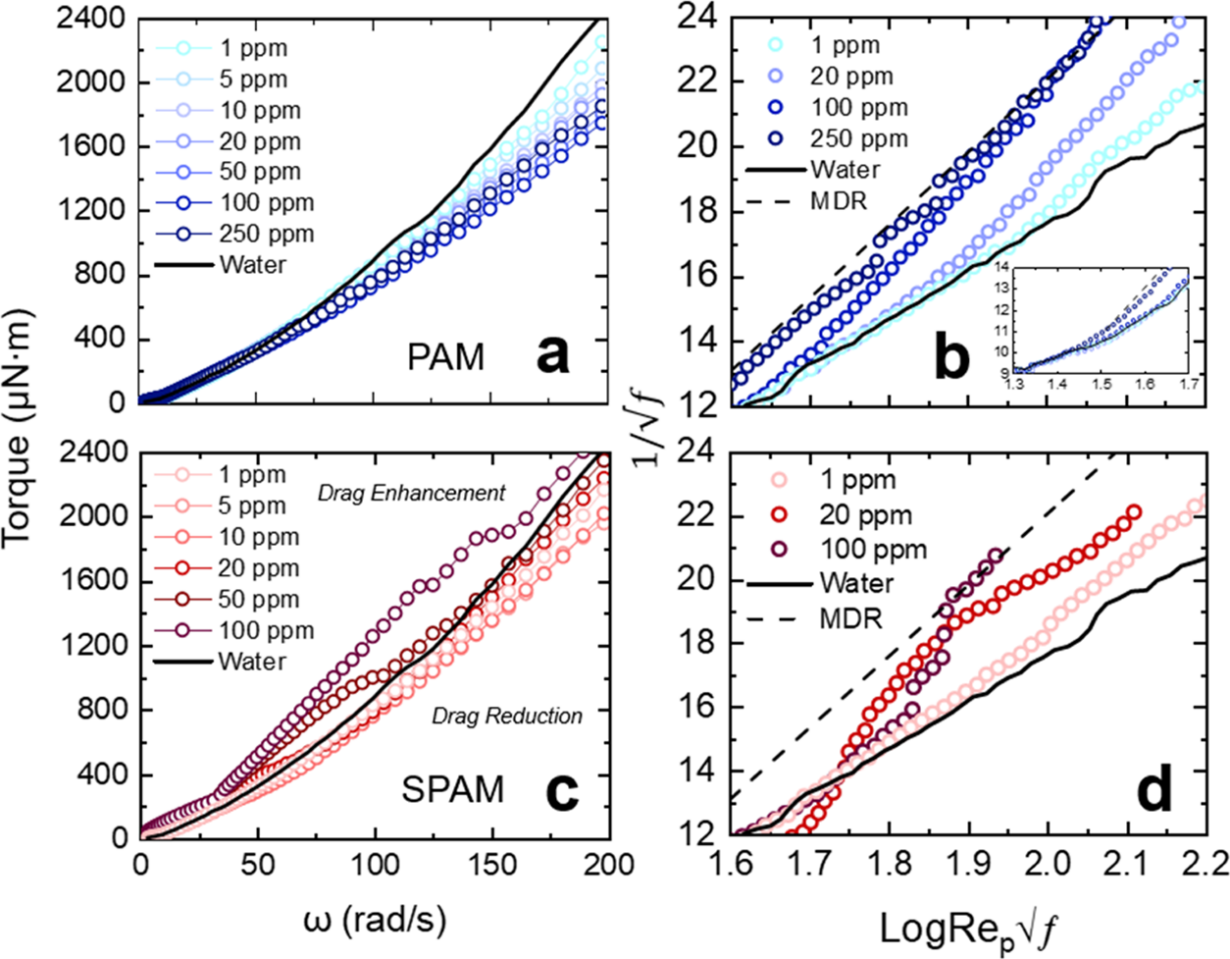
Fluid drag reduction by PAM (a, b) and SPAM (c, d) as a function of the polymer concentration. Panels (a) and (c) compare the raw data of torque against rotational speed. Panels (b) and (d) compare the dimensionless data plotted in Prandtl–von Karman coordinates for the condition Log *Re*_p_√*f* ≥ 1.6. The dashed lines (b, d) show the empirically determined maximum drag reduction (MDR), which was estimated using a least-squares linear regression approach, taking the form of eq 6 for the data of 500 < *c* < 750 ppm PAM. Further details are provided in Figure S2 of the Supporting Information.

Figure 4 shows typical drag reduction data for PAM (Figure 4a,b) and SPAM (Figure 4c,d) at increasing polymer concentrations in Milli-Q water. All data is compared to Milli-Q water only. When considering the raw data of rotational speed and torque, for PAM, at low rotational speeds (<10 rad/s) in the laminar flow regime, the polymer concentration-torque data superimposes the Milli-Q water baseline, which is characteristic for a Newtonian fluid and consistent with Bizotto and Sabadini et al.,^26^ who studied PEO and PAM drag-reducing fluids. The flow regime becomes unstable with increasing rotational speed and the measured torque values for the polymeric fluids diverge from the Milli-Q water baseline at a critical rotational speed, ω = ω_crit_, with the measured torque found to be lower at higher polymer concentrations, except at 250 ppm PAM, where a higher torque value was measured compared to 50 and 100 ppm. This inconsistency is magnified for SPAM (Figure 4c), with torque values mostly exceeding those of Milli-Q water in both the stable and unstable flow regimes. In the stable regime, this is partly attributed to the high fluid viscosity of the non-Newtonian SPAM fluids, shifting the transition to unstable flow and thus the onset of drag reduction, similar to the study of Dutcher and Muller,^30^ who attributed the shift in flow regime to be governed by weak fluid viscoelasticity. Although the flow is unstable, for SPAM concentrations of 50 and 100 ppm, the measured torque remains to exceed that of Milli-Q water over a wide range of rotational speeds, which may be interpreted as an apparent drag enhancement. Drag enhancement has also been observed in pressure-driven flows when using extended polymers and operating at low shear rates (low *Re*) below the onset of drag reduction.^43,44^ In contrast to pipe flow, defining drag reduction by rotational rheometry (low *Re*), as shown in this study, is less trivial.

The region of drag reduction (Log *Re*_p_√*f* >1.6) plotted in Prandtl–von Karman coordinates is shown in Figure 4b (PAM) and Figure 4d (SPAM). On the semi-log plot, the value of 1/√*f* increases with *Re*_p_, and for PAM, the data of 1/√*f* diverges from the Milli-Q water baseline while exhibiting a type A drag reduction response. At 1 ppm PAM, baseline divergence is seen at Log *Re*_p_√*f* = 1.9, but for 250 ppm, divergence occurs at a lower value, Log *Re*_p_√*f* = 1.42, very close to the onset of unstable flow, Log *Re*_p_√*f* = 1.29; hence, the onset of drag reduction occurs at a lower critical *Re*_p_ for higher polymer concentrations, which is consistent with other studies.^45,46^ For Log *Re*_p_√*f* > 1.8, the magnitude of drag reduction increases with increasing polymer concentration. However, at Log *Re*_p_√*f* = 2.0, the polymeric friction lines for 100 and 250 ppm PAM approach the maximum drag reduction asymptote (MDR, dashed line in Figure 4b), with the slope of the polymeric friction line at 250 ppm attaining similar values to the MDR line. Hence, further increases in polymer concentration produce no discernible effect on the drag reduction. Such behavior is more consistent with drag reduction in pipes,^47,48^ and underlines the need to accurately account for fluid viscosity when studying drag reduction using rotational rheometry with a small annular gap.

When accounting for the correct fluid viscosity to calculate *Re*_p_ (*Re* based on the rotational velocity and fluid viscosity, see eq 3), the 250 ppm PAM fluid shows drag reduction and not drag enhancement. For SPAM (Figure 4d), the effect of polymer concentration on drag reduction performance is less clear. At low *Re* (Log *Re*_p_√*f* ≲ 1.85), drag reduction increases in the following order: 20 > 100 > 1 ppm, while at higher *Re* (Log *Re*_p_√*f* ≳ 1.85), the order changes to 100 > 20 > 1 ppm and is more consistent with PAM fluids. This highlights the nonlinear response of SPAM (also seen in Figure 4c), which is attributed to mild fluid hysteresis at low *Re*_p_ and at concentrations ≥5 ppm.^49^ Similar hysteresis has been observed for polymers of high elasticity^30,50^ and is attributed to polymer conformational hysteresis.^51^ It is noted that the PAM fluids showed no hysteresis, even at the highest concentration of 750 ppm. The relevance of this effect is discussed with reference to Figure 9b.

**Figure 5:**
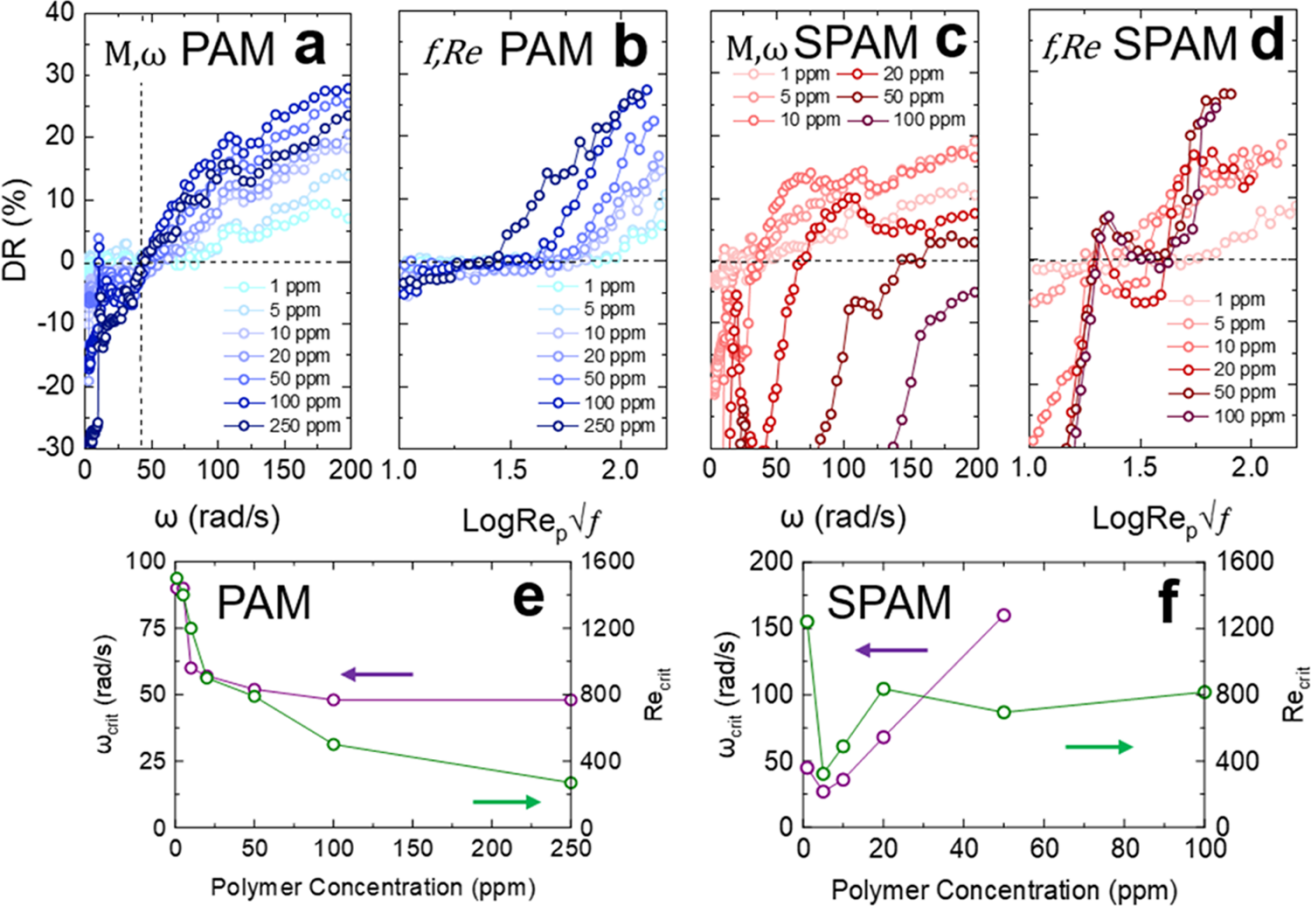
Polymer-induced drag reduction calculated using eq 4, where the variable (***x***) is taken to be the measured torque (a, c) or 1/√*f* (b, c) for polymers PAM (a, b) and SPAM (c, d). The effect of polymer concentration on the onset of drag reduction defined as the critical rotational speed (ω_crit_) and Reynolds number (*Re*_crit_) for PAM (e) and SPAM (f).

The calculated drag reductions (eq 4) based on torque and 1/√*f* are shown in Figure 5 for PAM (Figure 5a torque; Figure 5b 1/√*f*) and SPAM (Figure 5c torque; Figure 5d 1/√*f*). If the fluid viscosity were not considered (DR based on the torque), then the added polymer is seen to promote drag enhancement at low rotational speeds, which eventually decays as the rotational speed increases. As previously discussed, this is attributed to the high viscosity of the fluids, which is more prominent in SPAM than PAM. As such, at the highest SPAM concentrations, an apparent drag enhancement is observed at all rotational speeds; see Figure 5e,f for the comparison of the critical rotational speed (ω_crit_) to induce drag reduction. For PAM, ω_crit_ becomes independent of polymer concentration; however, the effect of polymer concentration on ω_crit_ remains strong for SPAM over the studied concentration range. Accounting for the changes in fluid viscosity with polymer concentration (Log *Re*_p_√*f*), the drag reduction response of the PAM fluids is corrected, and the fluids exhibit negligible drag enhancement (within the measurement noise) before a definitive drag reduction regime is observed. However, for the SPAM fluids, a strong drag enhancement remains for the highest polymer concentrations, which is attributed to an incorrect interpretation of *Re*_p_ when the flow regime is laminar, i.e., the condition of η_∞_ is not satisfied. It is noted that the apparent drag reduction with increasing *Re*_p_ appears to oscillate. The first increase in DR% is attributed to an extension of the laminar regime for high-viscosity fluids (i.e., *Ta* is inversely proportional to the fluid viscosity; hence the onset of flow instabilities is delayed), and thus the true onset of drag reduction is taken as the second intercept of DR% = 0, and thereafter, a constant enhancement of DR% was observed with increasing Log *Re*_p_√*f*. Based on this interpretation of the data, the *Re*_crit_ for PAM decreased with increasing polymer concentration, while for SPAM, the *Re*_crit_ fluctuated before being independent of polymer concentration when *c* ≥ 20 ppm. This response would not be consistent with type A drag reduction but may suggest a type B response, where the critical transition is less affected by the polymer concentration.^52^

To compare the drag reduction performance of PAM and SPAM, it is desirable to normalize the polymer concentration by the polymer volume fraction, which requires an accurate measure of the intrinsic viscosity.^53,54^ With SPAM being a polyelectrolyte, its reduced viscosity does not vary linearly with polymer concentration, and hence it is not possible to attain a reliable measure of the intrinsic viscosity in salt-free solutions. Therefore, the method of Little et al.^55^ was followed which normalizes the concentration by the intrinsic concentration (*c*_int_) of the polymer solution. The *c*_int_ was defined by Virk et al.^16^ as, , where DR_max_ is the theoretical maximum drag reduction and  is the intrinsic drag reduction. Plots of *c*/DR against c to determine *c*_int_ for both polymers are provided in Figure S3 of the Supporting Information.

**Figure 6:**
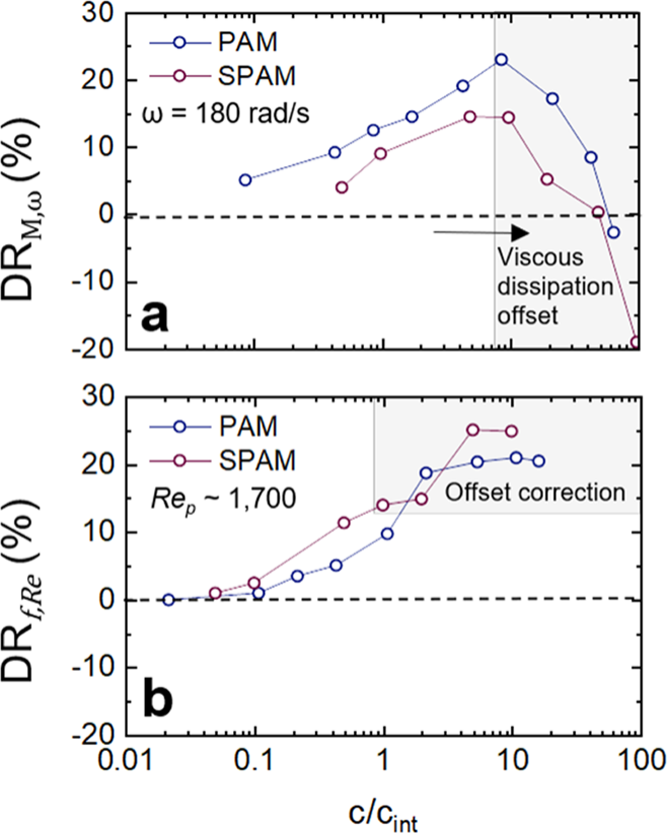
PAM and SPAM polymer drag reduction as a function of *c*/*c*_int_, where *c* is the polymer concentration (ppm) and *c*_int_ is the intrinsic concentration of the polymer fluid. The data is shown for both an equivalent rotational speed (a) and shear Reynolds number (*Re*_p_) (b). The gray-shaded regions represent the same data in both panels (a) and (b) but are included to highlight the reduction in DR% in panel (a) and the plateau of DR% in panel (b).

Figure 6a compares the DR% (difference in torques at the same rotational speed, 180 rad/s) as a function of *c*/*c*_int_, with the trend being consistent for both polymer fluids, and a maximum DR% was found when *c*/*c*_int_ ∼ 8, indicating the onset of a region influenced by the increasing fluid viscosity. When compared at constant *Re*_p_ (differences in friction factors) (Figure 6b), the DR% for both polymers increases with *c*/*c*_int_ before plateauing at the highest values of *c*/*c*_int_. In this region, SPAM is found to be a more efficient drag reducer than PAM, with a DR_max_ of 27.6% compared to 22.3% for PAM, and having a lower intrinsic concentration, see Table 1 for all values of DR_max_, *c*_int_, and . Therefore, to achieve equivalent values of DR%, the effective concentration of SPAM is less than PAM and confirms the polymer to be more efficient in promoting fluid drag reduction, with the difference in performance attributed to the greater apparent size of the anionic SPAM relative to the nonionic PAM.

**Table 1:** Parameters for a

polymer	DR_max_ (%)	% deviation from actual DR_max_	*c*_int_ (ppm)	
PAM_ω_	25.0	8.0	11.7	2.14
SPAM_ω_	16.2	10.7	1.0	15.77
PAM_Re_	22.3	5.8	46.5	0.48
SPAM_Re_	27.6	8.9	10.1	2.73

a The % deviation DR_max_ is the difference between the theoretical DR_max_ and measured DR_max_.

### Stability of Drag Reduction

**Figure 7:**
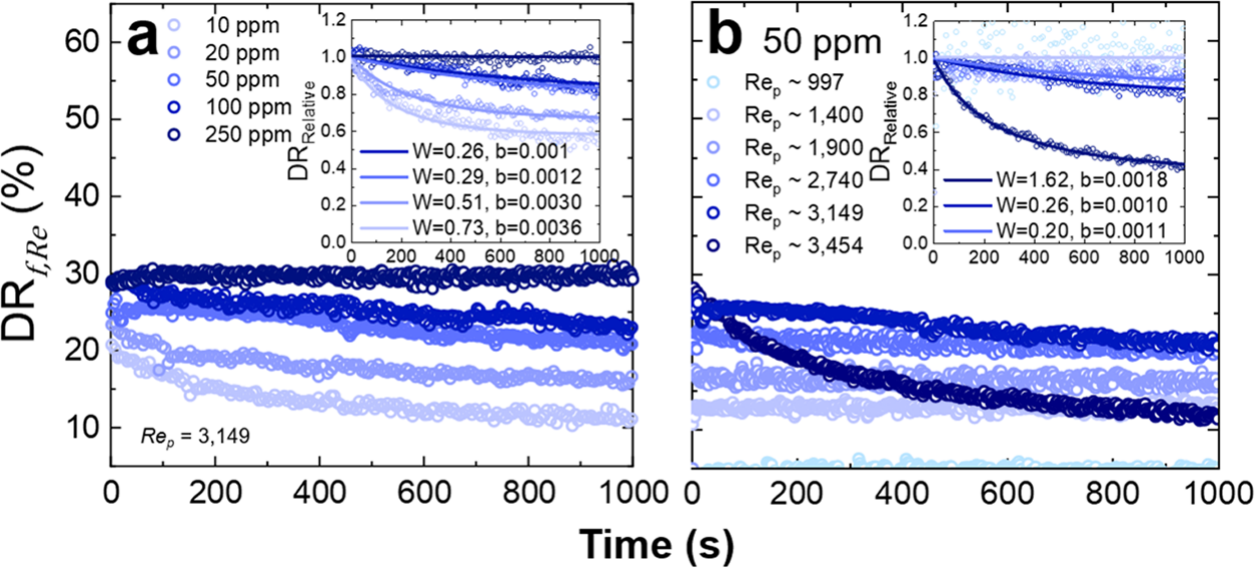
Drag reduction stability for PAM as a function of (a) increasing polymer concentration and at a constant *Re* and (b) at a fixed polymer concentration (50 ppm) and increasing *Re*_p_. All DR% values were calculated based on the friction factor. Insets show the relative change in drag reduction (DR_Relative_) with time. Equation 8 is used to fit the DR_Relative_ data, and the fitting parameters *W* and *b* are provided in the inset graphs.

Along with the maximum drag reduction, the shear stability should also be considered. From eq 4, and taking *x* as the friction factor (eq 2), the drag reduction with time is shown as: (i) a function of increasing polymer concentration at a constant *Re*_p_; and (ii) at a fixed polymer concentration and increasing *Re*_p_, see Figure 7 for PAM. The inset data in Figure 7 shows the relative change in DR from *t* = 0 (DR_Relative_ = DR_*t*_/DR_0_) and is fitted using a least-squares regression model that describes the loss in drag reduction with time, which is given by^56^
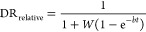
8where *W* and *b* are fitting parameters that describe the shear stability and decay rate, respectively.

For all PAM concentrations shown in Figure 7a, the flow regime is unstable at *Re*_p_ = 3149. With increasing polymer concentrations the relative stability of drag reduction increases as the turbulent fluid stresses become increasingly modulated. Such behavior is consistent with previous observations.^35^

At a fixed PAM concentration and increasing *Re*_p_ (Figure 7b), the relative change in drag reduction showed a weak dependence on *Re*_p_; only the highest *Re*_p_ values lead to measurable losses in DR. At low *Re*_p_, the fluid stress intensity is lower; hence polymer stretching is weakened, which leads to an apparent increase in polymer stability. At higher *Re*_p_ (*Re*_p_ > 3000), the strong interaction between the high-molecular-weight polymer and fluid instabilities imposes greater stress on the extended polymer chains, likely causing the linear polymer chains to undergo chain scission, reducing the molecular weight below a critical threshold needed to induce the DR_max_. As shown in the inset of Figure 7b, only at *Re*_p_ ∼ 3454 is the relative change in drag reduction significant, decreasing by almost 60% within 1000 s. At this flow condition, the initial apparent shear stress is 8.94 Pa compared to 6.81 Pa for *Re*_p_ ∼ 2740, where the relative change in drag reduction was <8%.

**Figure 8:**
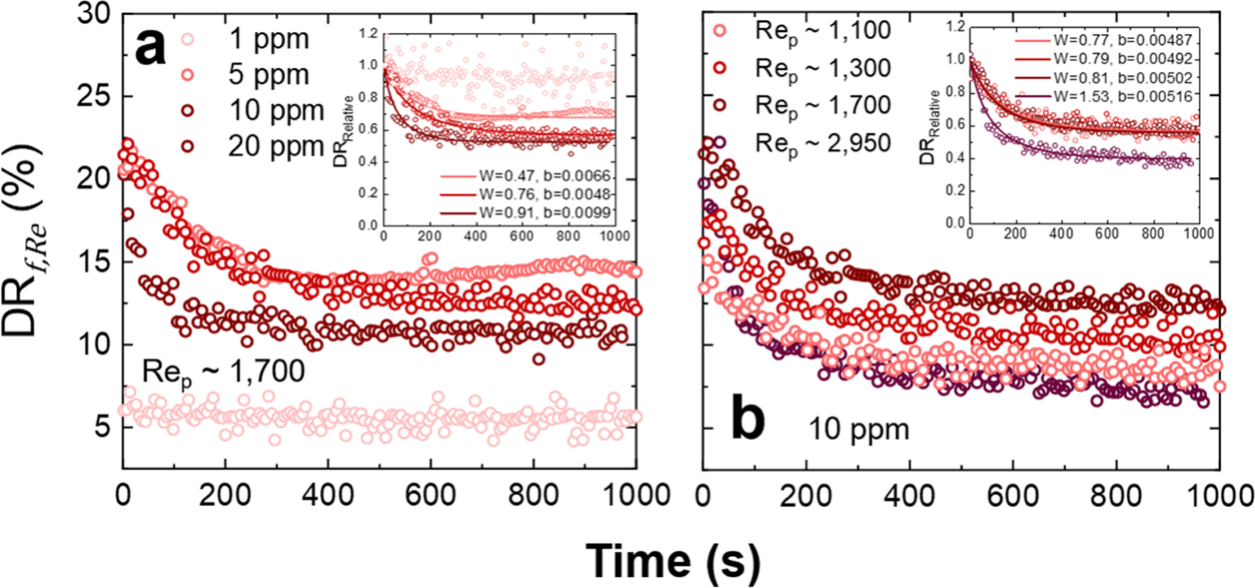
Drag reduction stability for SPAM as a function of (a) increasing polymer concentration and at a constant *Re* and (b) at a fixed polymer concentration (10 ppm) and increasing *Re*_p_. All DR% values were calculated based on the friction factor. Insets show the relative change in drag reduction (DR_Relative_) with time. Equation 8 was used to fit the DR_Relative_ data, with the fitting parameters *W* and *b* being provided in the inset graphs.

The drag reduction stability of SPAM as a function of polymer concentration and *Re*_p_ are shown in Figure 8. Due to the strong non-Newtonian fluid response, the highest concentration was limited to 20 ppm where the relative infinite shear viscosity of the polymer fluid was 1.08 × 10^–3^ Pa s. Unlike PAM, the effect of polymer concentration on DR loss is less clear. At 1 ppm, the DR% is low (∼5–7%) and likely contributes to the high apparent stability of the polymer with time. With increasing polymer concentration, the decay rate of drag reduction also increased. All polymer fluids appeared to degrade rapidly within the first 400 s of shearing; thereafter, the polymer fluids were stable. Although the range of concentrations was small, a faster loss of drag reduction at higher SPAM concentrations is counterintuitive and points to an influence of the semi-dilute regime and high occupied volume of the polymer chains in the fluid when measured using the rheometer method. Furthermore, for some experimental conditions, the stress applied was consistent with that used for the PAM tests, and hence those differences in apparent stability are independent of the fluid stress.

When increasing *Re*_p_ (1100 < *Re*_p_ < 2950) at a fixed polymer concentration (Figure 8b), the loss of drag reduction weakly depends on *Re*_p_. For *Re*_p_ between 1100 and 1700, the relative loss of drag reduction was very similar and only at *Re*_p_ ∼ 2950 was the rate of loss and the magnitude of drag reduction loss increased further. Although not commonly observed, a similar response of faster drag reduction loss at higher polymer concentration has been reported by Bizotto and Sabadini^26^ studying extended polyacrylamide. The authors attributed the behavior to the low optimum polymer concentration for DR_max_ and the high fluid viscosity, which in the current study may not be valid since 10 ppm SPAM and 250 ppm PAM have almost equal fluid viscosities (η_∞_) but undergo contrasting drag reduction losses. Moreover, the apparent rapid drag reduction loss of SPAM may be attributed to a time effect, as indicated by Pereira et al.,^45^ who showed that polymer (xanthan gum) de-aggregation can influence the apparent drag reduction stability. Gentle pre-shearing of the test fluids prior to measurement led to improved drag reduction stability (removed the rapid decay in drag reduction loss), which the authors attributed to de-aggregation of polymer chains in solution.

**Figure 9:**
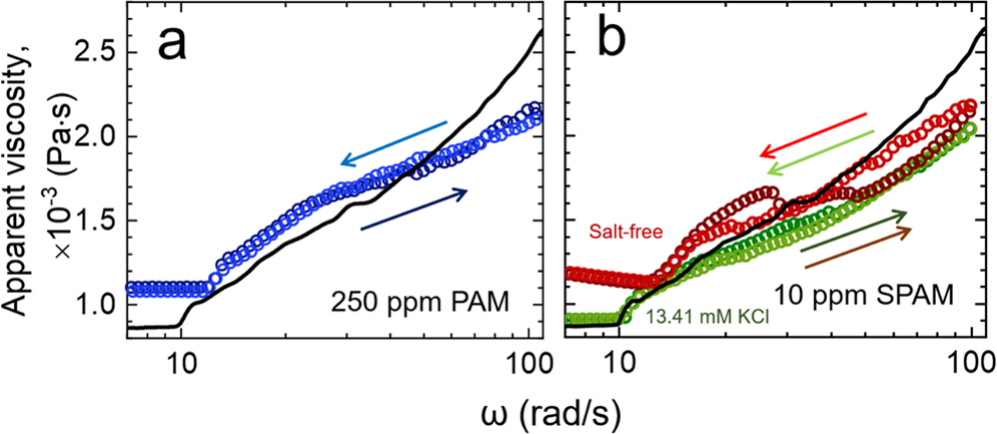
Rotational speed (ω, rad/s) flow sweep tests for (a) 250 ppm PAM and (b) 10 ppm SPAM in Milli-Q water and 13.41 mM KCl. The solid line is water.

To assess if time-dependency was important, 250 ppm PAM and 10 ppm SPAM (fluids of almost equivalent infinite shear fluid viscosities) were subjected to shear ramp tests, as shown in Figure 9. For 250 ppm PAM (below c*), negligible hysteresis was observed in the nonlaminar flow regime, and the slight reduction in fluid viscosity (2 × 10^–5^ Pa s) before and after shearing may correspond to a small decrease in the average polymer molecular weight by shear-induced polymer chain scission but is noted to be within the measurement error. The hysteresis loop for 10 ppm SPAM (semi-dilute regime, above c*) is more pronounced in the nonlaminar flow regime, and a crossover in the loop was observed in the region of emerging drag reduction. In the laminar flow regime, the fluid viscosities before and after shearing were equivalent, indicating no apparent change in the average molecular weight of the polymer. The contrasting hysteresis response between the two fluids suggests that the SPAM fluid does not undergo substantial polymer degradation, and the apparent rapid loss of drag reduction is likely due to the hysteresis (time effect) response of the fluid. The hysteresis response of SPAM could be diminished by increasing the salt (KCl) concentration. By neutralizing the charge on SPAM the polymer behaves similarly to the nonionic PAM (Figure 9b) and undergoes reduced loss of drag reduction under prolonged shearing (Figure S4). However, this behavior would contradict the general understanding that adding salt to charged polymers decreases the stability of drag reduction.^35^

These observations further support the understanding that the apparent rapid loss of drag reduction for SPAM (Figure 8), when measured using the double-gap concentric cylinder geometry, is not due to polymer degradation but a predominantly shear/time-dependent effect when the polymer concentration is in the semi-dilute regime.

### Comparison of Rheometry and Pipe Flow Data

**Figure 10:**
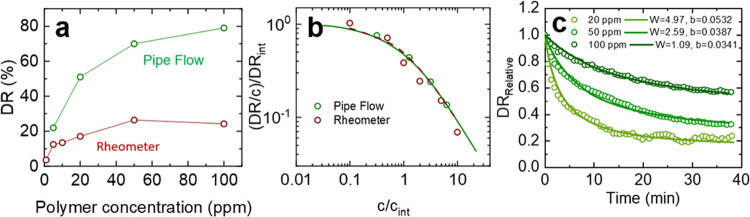
(a) Pipe flow and rheometry DR% for SPAM as a function of the polymer concentration. The flow conditions for the double-gap geometry and pipe flow were *Re* ∼ 1700 and *Re*_p_ ∼ 97,000 (5 ppm SPAM) to ∼51,000 (100 ppm SPAM). (b) Normalized DR/c as a function of the normalized polymer concentration for both pipe flow and rheometry geometries. The determination of DR_int_ and *c*_int_ are given in Figure S5 of the Supporting Information. The fitting parameters for the data are provided in Table 2. (c) Drag reduction stability for SPAM as a function of the polymer concentration and at a constant volumetric flow rate. The Brostow model (eq 8) was used to fit the data with the parameters *W* and *b* provided in the figure.

Although rotational rheometry can be used to rapidly screen polymers for drag reduction, it is important that the characteristic behavior is consistent with pipe flow. Figure 10a compares the drag reduction performance of SPAM as measured by rotational rheometry and pipe flow, with the flow conditions for both setups being *Re*_p_ ∼ 1700 (*Ta* < 7.7 × 10^5^, turbulent Taylor vortex regime) and *Re*_p_ from ∼51,000 to ∼97,000 (dependent on the polymer concentration). While the trends in DR% with increasing SPAM concentration were similar (Figure 10a), the magnitude of drag reduction was vastly different, which could be attributed to the differences in *Re* and the associated flow regimes. While lower flow rates for the pipe flow were considered, harmonic instabilities in the pressure response meant lower flow rates were not possible; as such, 80 L/min was taken as the lowest flow rate. At the test conditions, the rheometer data indicated an optimum polymer concentration between 50 and 100 ppm, whereas, for the pipe flow, the optimum concentration was slightly above 100 ppm as the DR% asymptotes to the MDR. We attribute this difference to the time effect that is more apparent in the rheometer data than in the pipe flow data. At higher SPAM concentrations, the time effect is more severe (Figure 8a), which slightly offsets the drag reduction effect.

Taking the approach of Virk et al.,^16^ the DR% from both pipe flow and rheometry experiments can be compared when plotting  against *c*/*c*_int_. As shown in Figure 10b, the two data sets are in good agreement (the experimental fits take the form of , where *k* describes the polymer–solvent interaction, and all fitting parameters are provided in Table 2), which means that the polymer concentration dependence on drag reduction is consistent between the two geometries. Moreover, the intrinsic concentrations (*c*_int_) for the rheometer and pipe flow geometries were 10.1 and 15.9 ppm, respectively, confirming that the polymer concentrations to achieve half DR_max_ were similar. However, as previously noted, caution should be taken when assessing the drag reduction stability of non-Newtonian semi-dilute polymers using the rheometer technique. This is because, when measuring the effect of SPAM concentration on drag reduction loss, the pipe flow data was more consistent with the common understanding that drag reduction is more stable at higher polymer concentrations.^35^ It is also noted that the loss of drag reduction occurs over tens of minutes rather than a few minutes (Figure 8), suggesting that the response is not a viscoelastic time effect as seen in the rheometer, but is more likely to be drag reduction loss due to a change in the average molecular weight of the polymer. With higher fluid shear stresses in pipe flow (wall shear stress up to ∼60 Pa) compared to the rheometer (shear stress up to ∼8 Pa), polymer chain scission is expected to be more severe.

**Table 2:** Fitting arameters from Figure 10b for both rheometer and pipe flow data^a^

geometry	DR_max_ (%)	% deviation from actual DR_max_	*c*_int_ (ppm)		*k*
rheometer	27.6	8.9	10.1	2.7	1
pipe loop	92.5	14.6	15.9	5.8	1

a The % deviation DR_max_ is the difference between the theoretical DR_max_ and measured DR_max_.

## Conclusions

The drag reduction performance of high-molecular-weight polymers (PAM and SPAM) was measured using a shear rheometer with double-gap concentric cylinder geometry. This method is ideal for chemical screening as it needs small sample volumes and tests can be rapidly run. However, the performance characteristics as measured by rheometry are not often correlated to pipe flow data, and this study highlights features of the rheometry method that must be correctly interpreted to ensure reliable performance characteristics are described.

Two high-molecular-weight (several MDa) polymers, nonionic PAM and anionic sulfonated PAM, were selected as they provided contrasting rheology. The high c* for PAM meant the test fluids were Newtonian and dilute, while the low c* (semi-dilute) for SPAM led to non-Newtonian, shear-thinning behavior. For the rheometry method with Taylor instabilities, the performance of DR based on the raw data increased with polymer concentration up to *c*/*c*_int_ ∼ 8, but at higher concentrations the DR% appeared to fall due to a greater influence of the polymeric fluid viscosity on the measured torque. To correctly describe the data, the raw data was analyzed by calculating the friction factor and the *Re* based on the infinite shear viscosity of the fluid to compare DR% at equivalent *Re*_p_. The maximum drag reduction of SPAM exceeded that of PAM at *Re*_p_ ∼ 1700, with the improved performance attributed to the slightly higher Mw and apparent size of the charged polymer. With extended shearing, the drag reduction loss of PAM was generally consistent with literature findings,^2,45^ in that higher polymer concentrations extended the time of drag reduction and higher *Re*_p_ increased the rate of drag reduction loss. The drag reduction stability of SPAM showed negligible effects of polymer concentration and *Re*_p_, with a significant loss of drag reduction occurring within 200–300 s. For SPAM, shear sweep tests revealed a hysteresis (viscoelastic effect) that contributes to the apparent fast decay in drag reduction performance. This is likely a consequence of the polymer concentration being in the semi-dilute regime and the non-Newtonian fluid response. In pipe flow, the effect is not apparent due to the high stresses imposed on the fluid, with the loss of drag reduction occurring over tens of minutes and the stability being dependent on the polymer concentration, leading to a behavior more consistent with the mechanism of polymer degradation via shear-induced polymer chain scission. While the stability data for non-Newtonian polymeric fluids in the semi-dilute regime is less reliable when measured by the double-gap geometry, the relative scaling of drag reduction with polymer concentration was found to be reasonably consistent between the two flow geometries.

## Supporting Information Available

Carreau–Yasuda model parameters and viscosity values for SPAM in salt-free and salt solutions and PAM (Table S1); specific viscosity η_sp_ as a function of polymer concentration (Figure S1); drag reduction data for PAM at 500 and 750 ppm plotted in Prandtl–von Karman coordinates used to approximate the MDR asymptote for the double-gap concentric cylinder geometry (Figure S2); drag reduction for PAM and SPAM at equivalent rotational speeds of 180 rad/s (a, b) and equivalent Reynolds number of ∼1700 (c, d) (Figure S3); drag reduction stability for 10 ppm in KCl electrolyte solutions of increasing concentration, 0 ≤ *c*_KCL_ ≤ 13.41 mM (Figure S4); drag reduction for SPAM in pipe flow (Figure S5) (PDF)
