# Supplementary material for: Polymer-Induced Drag Reduction in Dilute Newtonian and Semi-Dilute Non-Newtonian Fluids: An Assessment of the Double-Gap Concentric Cylinder Method
Source: Ind Eng Chem Res. 2022 Jul 20;61(30):11197–208. doi: 10.1021/acs.iecr.2c00899 (PMC9354081; doi:10.1021/acs.iecr.2c00899)
Supplement: Supplementary file 1 — ie2c00899_si_001.pdf [file ie2c00899_si_001.pdf]

Supplementary Information

Polymer-induced drag reduction in dilute Newtonian and semi-dilute non-Newtonian fluids: An assessment of the double-gap concentric cylinder method

Stefanos Michaelides[1], Kotaybah W. Hashlamoun[2], Thibaut Charpentier[1], Gregory de Boer[3], Paul Hunt[4], Helen Sarginson[4], Claire Ward[4], Nashaat N. Nassar[2], Mark C.T. Wilson[3] and David Harbottle[1*]

[1]School of Chemical and Process Engineering, University of Leeds, Leeds, LS29JT, UK
[2]Department of Chemical and Petroleum Engineering, University of Calgary, Alberta, T2N1N4, Canada
[3]School of Mechanical Engineering, University of Leeds, Leeds, LS29JT, UK
[4]CRODA Europe Ltd, Goole, DN149AA, UK
E: D.H., d.harbottle@leeds.ac.uk

S1 – Shear Rheology. The Carreau-Yasuda model was used to fit the shear viscosity data of SPAM in salt-free Milli-Q water in the concentration range of 10 < c < 1,000 ppm. All PAM samples were Newtonian for the concentration range of 1 < c < 2,500 ppm and only exhibited weak shear thinning behavior for c $\geq$ 5,000 ppm. It was therefore assumed that the infinite shear viscosity of PAM samples was equal to viscosity at the shear rate just before the onset of Taylor instabilities, with the exception of c = 5,000 and 7,500 ppm, where η_∞ was determined by fitting the Carreau-Yasuda model. All the parameters for the Carreau-Yasuda model are provided in Table S1.

Table S1. Carreau-Yasuda model parameters and viscosity values for SPAM in salt-free and salt solutions, and PAM.

Polymer	C (ppm)	η_0 (mPa·s)	η_∞ (mPa·s)	k	n	a
**SPAM**	1	-	0.88	-	-	-
	2	-	0.895	-	-	-
	3	-	0.935	-	-	-
	5	-	0.975	-	-	-
	10	-	0.947	0.37	0.093	0.28
	20	-	1.08	2.19	0.11	0.23
	50	-	1.58	42.53	0.28	5.13
	100	510	1.84	21.0	0.25	1.58
	250	1520	1.91	37.69	0.29	4.24
	500	2870	1.82	39.85	0.31	2.78
	1000	3780	1.93	19.58	0.31	1.74
**SPAM (KCl, g/L)**	10 (0.01 g/L)	-	0.913	-	-	-
	10 (0.1 g/L)	-	0.902	-	-	-
	10 (1 g/L)	-	0.895	-	-	-
**PAM**	1	0.862	0.862	-	-	-
	5	0.863	0.863	-	-	-
	10	0.888	0.888	-	-	-
	15	0.891	0.891	-	-	-
	20	0.899	0.899	-	-	-
	50	0.921	0.921	-	-	-
	100	0.985	0.985	-	-	-
	250	1.10	1.10	-	-	-
	500	1.28	1.28	-	-	-
	75	1.57	1.57	-	-	-
	1000	1.66	1.66	-	-	-
	2500	4.19	4.19	-	-	-
	5000	15.44	6.64	0.022	0.52	1.16
	7500	39.81	9.81	0.025	0.38	0.93

S2 – Polymer overlap concentration (c*). For neutral polymers (PAM) and polyelectrolytes (SPAM) the overlap concentration (c*) is approximately the concentration at which the specific viscosity, $\eta_{sp}{\sim}1$.[1] The specific viscosity, η_{sp}, is given by:

$$\eta_{specific} = \frac{\eta_{zero\ shear\ polymer} - \eta_{solvent}}{\eta_{solvent}} \qquad \text{(SE1)}$$

For PAM there is a clear transition from the dilute to semi-dilute regimes at ~1,500 ppm (Fig. S1), which corresponds to a value of $\eta_{sp}{\sim}1$. As expected by the Zimm model,[1] the slope of the fit in the dilute regime for PAM is close to unity (~0.87), with an increase of slope in the semi-dilute regime. For SPAM there was no experimental method to determine c* due to an inability to determine the zero-shear viscosity in the concentration range of 1 < c < 50 ppm, due to torque limitations of the rheometer at very low shear rates (1/s). The method of Behra et al.[2] was followed by fitting η_{sp} and extrapolating to $\eta_{sp}{\sim}1$ only using those concentrations where the zero shear viscosity could be experimentally determined and fitted by the model. The slope of the fit was ~0.7 and close to the scaling of ~0.5 taken from the Rouse model.[1] Using this method, the c* of SPAM in Milli-Q water was ~0.01 ppm (Fig. S1). This is consistent with the general understanding that most polyelectrolytes in salt-free solution are in the semi-dilute regime.[1]

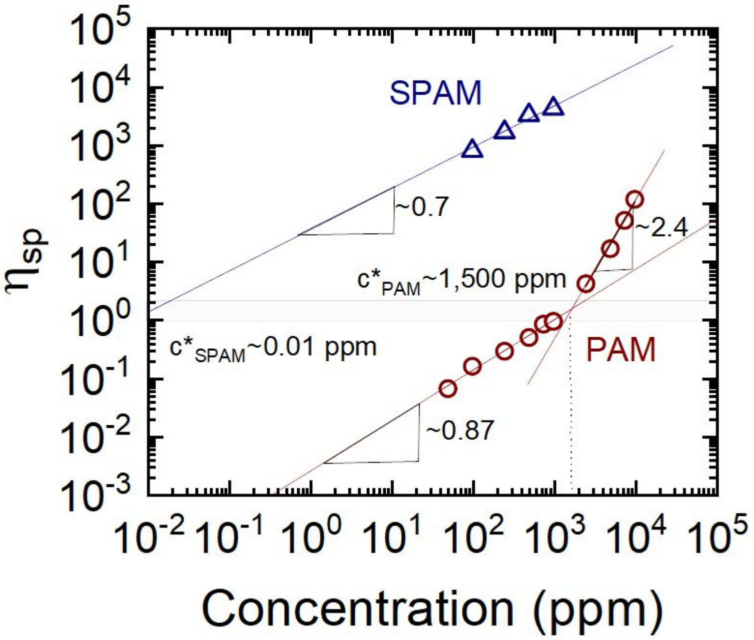

Figure S1. Specific viscosity $\boldsymbol{\eta_{sp}}$ as a function of polymer concentration. The grayed area indicates the region where $\boldsymbol{\eta_{sp}{\sim}1}$ for which c = c*.

S3 – Maximum DR asymptote. An alternative to the Virk equation,[3,4] the MDR asymptote was approximated for the double-gap concentric cylinder geometry, using PAM in the concentration range, 500 < c < 750 ppm. The least-square linear regression was obtained by the average best fit at those concentrations as $11.3 < \frac{1}{\sqrt{f}} < 23.5$ and $1.50 < Re\sqrt{f} < 2.03$. The approach is similar to that used by Rajappan and McKinley,[5] although polymer elasticity has not been considered in the current study. The least-square linear fit is given by $\frac{1}{\sqrt{f}} = 22.4 \log Re\sqrt{f} - 22.7$ (Fig. S2). A strong indication that MDR is attained is that for higher concentrations of PAM led to no discernible improvement in the DR% but rather an overlay of the polymeric friction data.

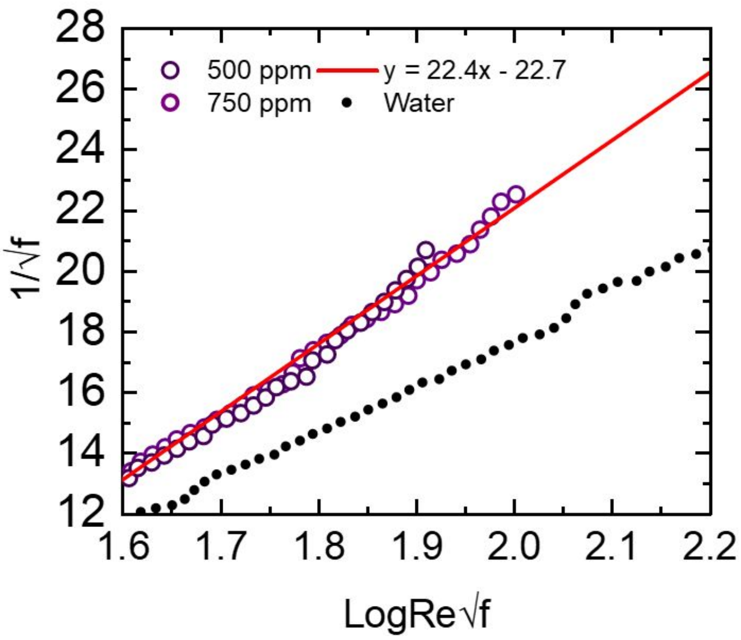

Figure S2. Drag reduction data for PAM at 500 and 750 ppm plotted in Prandtl-von Karman coordinates, used to approximate the MDR assymptote for the double-gap concentric cylinder geometry.

S4 – Determining the intrinsic concentration (c_{int}). Following the method of Little[6] to obtain a correlation of DR% with polymer concentration, the equation of Virk[3] can be simplified to:

$$\frac{c}{DR} = \frac{c_{int}}{DR_{max}} + \frac{c}{DR_{max}} \quad \text{(SE2)}$$

Equation SE2 shows a linear relationship between the quantities c/DR and concentration (Fig. S3). The intercept at c/DR = 0 provides the intrinsic concentration, c_{int}, from which the theoretical DR_{max} is determined.[6] Only data below the polymer concentration at which the viscous dampening begins to affect the fluid response has been considered.

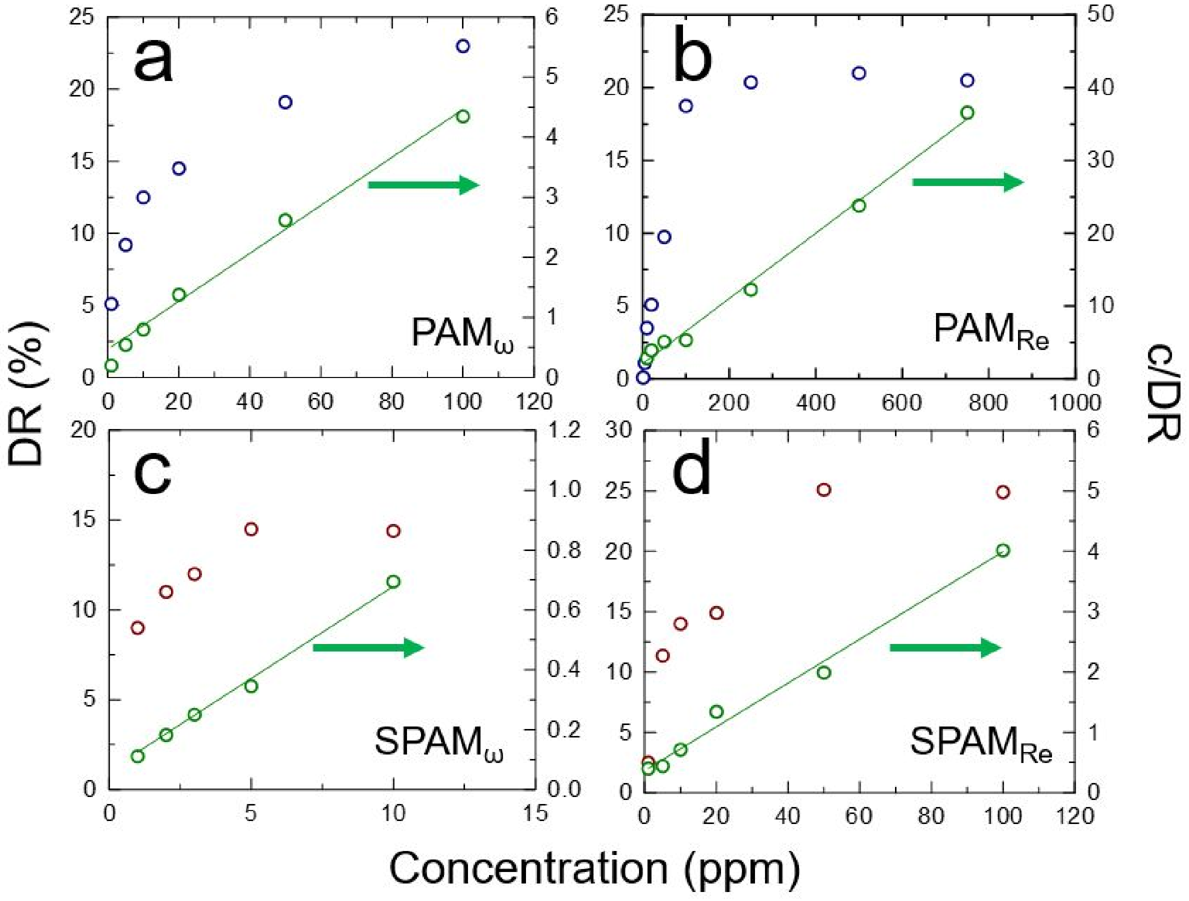

Figure S3. The drag reduction for PAM and SPAM at equivalent rotational speeds of 180 rad/s (a and b) and equivalent shear Reynolds number of ~1,700 (c and d). The second y-axis is c/DR based on the experimental DR at the respective concentrations. The lines represent Eq. SE2 for the concentrations, $c < c_{crit}$.

S5 – Drag reduction of SPAM with increasing salt concentration. The apparent shear stability of 10 ppm SPAM as a function of the KCl concentration are shown in Fig. S4. With increasing salt concentration, the DR% decreases and the apparent shear stability increases, with the effect attributed to the polymer coil collapsing to a globule at higher salt concentrations. Similar behavior was observed at 5 and 20 ppm SPAM (data not shown).

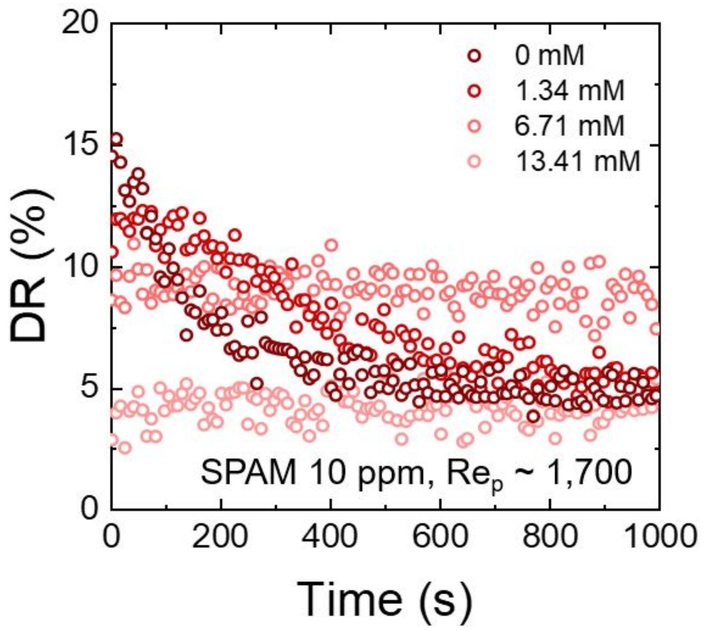

Figure S4. Time-dependent drag reduction for 10 ppm SPAM in KCl electrolyte solutions of increasing concentration, $0 \le c_{KCL} \le 13.4$ mM.

S6 – Drag reduction of SPAM in pipe flow. The drag reduction data of SPAM in pipe flow for concentrations, 5 < c < 100 ppm.

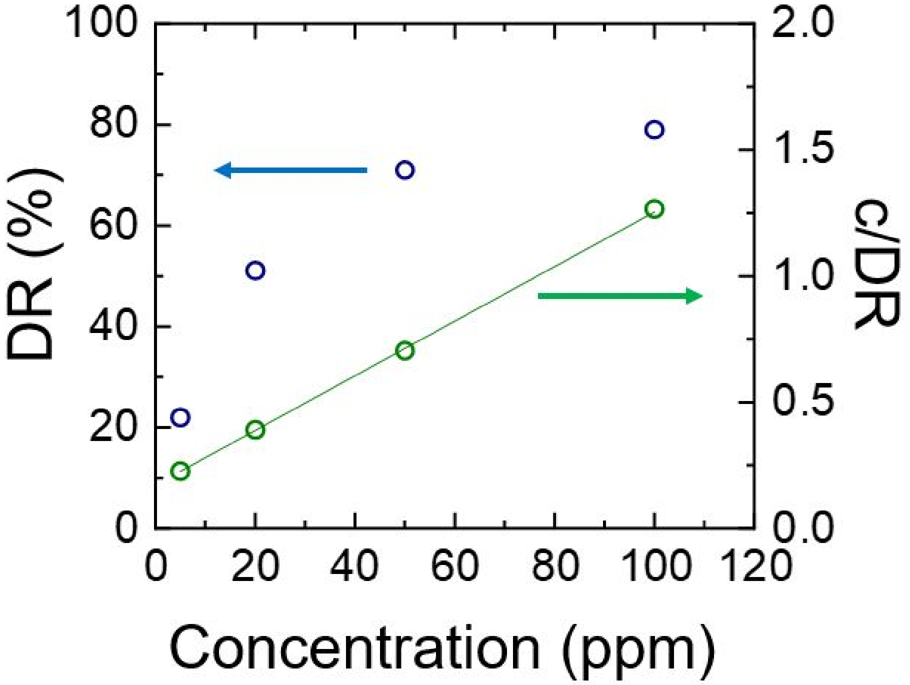

Figure S5. The drag reduction for SPAM in pipe flow. The second y-axis is c/DR based on the experimental DR at the respective concentrations. The line represents Eq. SE2 for concentrations, $c < c_{crit}$.

References

1. Colby, R. H. Structure and linear viscoelasticity of flexible polymer solutions: Comparison of polyelectrolyte and neutral polymer solutions. *Rheologica Acta* **49**, 425–442 (2010).
2. Behra, J. S. *et al.* Characterization of Sodium Carboxymethyl Cellulose Aqueous Solutions to Support Complex Product Formulation: A Rheology and Light Scattering Study. *ACS Appl. Polym. Mater.* **1**, 344–358 (2019).
3. Virk, P. S., Merrill, E. W., Mickley, H. S., Smith, K. A. & Mollo-Christensen, E. L. The Toms phenomenon: Turbulent pipe flow of dilute polymer solutions. *J. Fluid Mech.* **30**, 305–328 (1967).
4. Virk, P. S. Drag reduction fundamentals. *AIChE Journal* (1975). doi:10.1002/aic.690210402
5. Rajappan, A. & McKinley, G. H. Polymers and Plastrons in Parallel Yield Enhanced Turbulent Drag Reduction. *Fluids* **5**, 197 (2020).
6. LITTLE, R. C. *et al.* the Drag Reduction Phenomenon. Observed Characteristics, Improved Agents and Proposed Mechanisms. **14**, 283–296 (1975).
